# Epistatic Relationships in the BRCA1-BRCA2 Pathway

**DOI:** 10.1371/journal.pgen.1002183

**Published:** 2011-07-14

**Authors:** Ralph Scully

**Affiliations:** Department of Medicine, Harvard Medical School and Beth Israel Deaconess Medical Center, Boston, Massachusetts, United States of America; National Cancer Institute, United States of America

In modern molecular genetics, epistasis analysis is a tool for probing the relationship between two genes and, hence, between the two genes' products. In its broadest sense, an epistatic relationship exists when combinations of specific alleles of two or more genes generate a quantitative phenotype that differs from the simple addition of phenotypes associated with each individual allele. Many different types of gene–gene interaction could be characterized as epistatic. In its purest form, an allele *a* of gene *A* is said to be epistatic to (literally, “standing over”) allele *b* of gene *B* if both *a* and *b* individually confer a quantitative defect in a particular phenotype, the defect caused by *a* is more severe than *b*, and the phenotype of *ab* resembles that of *a*. In this case, the presence of allele *b* is irrelevant if allele *a* is present. Clear-cut genetic interactions of this type might indicate that there is an equally clear-cut biochemical interaction between the gene products, A and B. In the example outlined above, A might act on the same biochemical pathway as B and effectively control its activities.

Epistasis analysis has provided useful insights into the quite complex gene relationships that constitute the BRCA1/BRCA2 pathway of homologous recombination (HR). In the early days of work on BRCA1 and BRCA2, as their roles in HR were beginning to come into focus, several studies indicated that *Brca* null mice die in utero around E7.5 (*Brca1* ^−/−^) or E8.5 (*Brca2* ^−/−^). The two phenotypes were similar—cell cycle arrest accompanied by p53 activation and a chromosome breakage pattern with a predominance of chromatid-type aberrations. Ludwig et al. attempted an epistasis analysis of *BRCA1* and *BRCA2* mutation in the mouse and observed three *Brca1* ^−/−^ *Brca2* ^−/−^ embryos that died ∼E7.5 [1]. Although the statistical power of this experiment was low, it suggested that *Brca1* ^−/−^ might be epistatic to *Brca2* ^−/−^ and appeared to support the idea that BRCA1 and BRCA2 operate on a common biochemical pathway connected with organismal viability. More than a decade later, it is clear that that conclusion was basically correct; however, many interesting and important distinctions between the two genes have also come to light. Notably, the lethal phenotype of *BRCA1* null mice is largely suppressed by deletion of *53BP1* [2], [3].

Differences between BRCA1 and BRCA2 have also emerged from studies of their molecular function. BRCA2 associates stoichiometrically with Rad51 and has a key role in loading Rad51 onto single-stranded DNA that is created by double-strand break (DSB) resection, as a prelude to Rad51-mediated strand exchange. In contrast, BRCA1 functions in earlier steps of DSB repair, both by controlling DSB resection and by facilitating the transition from DSB resection to BRCA2-mediated Rad51 loading via BRCA1's and BRCA2's mutual interactions with PALB2/FANCN. BRCA1 participates in multiple distinct protein complexes, some of which function on chromatin and may regulate transcription.

In the clinical setting, *BRCA1*-linked breast cancers are more often of the “triple negative” basal type than are either sporadic or *BRCA2*-linked breast cancers, for reasons that are not well understood. Very likely, breast/ovarian cancer risk and the responsiveness of *BRCA*-linked cancers to poly(ADP) ribose polymerase (PARP) inhibitors are reflections of HR functions shared by *BRCA1* and *BRCA2*. The additional phenotypic “overlay” associated with *BRCA1*-linked cancers may reflect the impact of perturbing *BRCA1* functions that are not shared with *BRCA2*.

Despite this progress in understanding the functions of BRCA1 and BRCA2, little epistasis data regarding their relationship has been reported since the work of Ludwig et al. In this issue of *PLoS Genetics*, Qing et al. have used the genetically tractable chicken lymphoblastoid cell line DT40 to undertake such an analysis, focusing on *BRCA2* [4]. Their work sheds important light on the functional relationships between BRCA1, BRCA2, Rad51, and other HR mediators. First, by generating a (probable) null allele of *BRCA2*, they show that complete or near-complete loss of *BRCA2* function is compatible with cell viability, in dramatic contrast to the *Rad51* null phenotype, which is cell lethal in DT40 [5]. Thus, although BRCA2 loads Rad51 protein, *Rad51* must have cell viability functions that are independent of *BRCA2*. Combined deletion of *BRCA1* and *BRCA2* produced no additive impairment of cell growth. However, when the authors studied the impact of combined *BRCA1* and *BRCA2* mutations on sensitivity to DNA damaging agents, a more complex and nuanced picture emerged. In response to cis-platin, an agent that induces interstrand DNA cross-links and engages HR as an essential part of their repair, or to the PARP inhibitor olaparib, *BRCA2* null mutation appears to be epistatic to all HR mediator mutants the authors tested, including *BRCA1*. However, in the response to camptothecin, a topoisomerase I poison that induces DSBs at replication forks, the pattern was quite different. *BRCA1* null cells were found to be much more sensitive to camptothecin than *BRCA2* null cells and, surprisingly, the combined mutation produced an intermediate phenotype. Thus, loss of *BRCA2* partially ameliorates the *BRCA1* null hypersensitivity to camptothecin. This deviation from simple epistasis is important and not yet explained, but a reasonable starting assumption is that the different DNA structures generated by the three DNA damaging agents tested call to a different extent upon specific and distinct DNA repair functions of BRCA1 and BRCA2. In this regard, a gene might appear to be a “master regulator” of its peers in one setting and their “humble servant” in another.

Understanding the extent to which these complex genetic interactions are played out in tumorigenesis and in the response to different cancer therapies is an important goal of translational research. We should expect more surprises and insights to emerge from epistasis analysis of *BRCA*-linked cancer and cancer therapy in animal models.

## References

[1] Ludwig T, Chapman DL, Papaioannou VE, Efstratiadis A (1997). Targeted mutations of breast cancer susceptibility gene homologs in mice: lethal phenotypes of Brca1, Brca2, Brca1/Brca2, Brca1/p53, and Brca2/p53 nullizygous embryos.. Genes Dev.

[2] Bouwman P, Aly A, Escandell JM, Pieterse M, Bartkova J (2010). 53BP1 loss rescues BRCA1 deficiency and is associated with triple-negative and BRCA-mutated breast cancers.. Nat Struct Mol Biol.

[3] Bunting SF, Callen E, Wong N, Chen HT, Polato F (2010). 53BP1 inhibits homologous recombination in Brca1-deficient cells by blocking resection of DNA breaks.. Cell.

[4] Qing Y, Yamazoe M, Hirota K, Dejsuphong D, Sakai W (2011). The epistatic relationship between BRCA2 and the other RAD51 mediators in homologous recombination.. PLoS Genet.

[5] Sonoda E, Sasaki MS, Buerstedde JM, Bezzubova O, Shinohara A (1998). Rad51-deficient vertebrate cells accumulate chromosomal breaks prior to cell death.. Embo J.

